# Engineering a marine microalga *Chlorella* sp. as the cell factory

**DOI:** 10.1186/s13068-023-02384-2

**Published:** 2023-09-07

**Authors:** Xinping Gu, Ying Deng, Aoqi Wang, Qinhua Gan, Yi Xin, Kalyanee Paithoonrangsarid, Yandu Lu

**Affiliations:** 1 Single-cell BioEngineering Group, State Key Laboratory of Marine Resource Utilization in South China Sea, School of Marine Life and Aquaculture, Hainan University, Haikou, 570228 China; 2 Biochemical Engineering and Systems Biology Research Group, National Center for Genetic Engineering and Biotechnology, National Science and Technology Development Agency at King Mongkut’s University of Technology Thonburi, Bangkok, 10150 Thailand; 3 Hainan Provincial Key Laboratory of Tropical Hydrobiotechnology, Hainan University, Haikou, China; 4 Haikou Technology Innovation Center for Research and Utilization of Algal Bioresources, Hainan University, Haikou, China

**Keywords:** Marine microalgae, *Chlorella*, Transformation, Cell factory

## Abstract

The use of marine microalgae in industrial systems is attractive for converting CO_2_ into value-added products using saline water and sunlight. The plant nature and demonstrated industrial potential facilitate *Chlorella* spp. as excellent model organisms for both basic research and commercial application. However, the transformation method has not been developed in marine *Chlorella* spp., thus genetic engineering is hindered in exploiting the industrial potentialities of these strains. In this study, we provided a transformation protocol for the marine *Chlorella* strain MEM25, which showed robust characteristics, including high production of proteins and polyunsaturated fatty acids in multiple cultivation systems over various spatial–temporal scales. We showed that transformants could be obtained in a dramatically time-saving manner (comparable to *Saccharomyces cerevisiae*) with four functional proteins expressed properly*.* The transgenes are integrated into the genome and can be successfully inherited for more than two years. The development of a marine *Chlorella* transformation method, in combination with the complete genome, will greatly facilitate more comprehensive mechanism studies and provide possibilities to use this species as chassis for synthetic biology to produce value-added compounds with mutual advantage in neutralization of CO_2_ in commercial scales.

**Supplementary Information:**

The online version contains supplementary material available at 10.1186/s13068-023-02384-2.

## Introduction

Microalga-based carbon neutralization holds great promise for net zero or negative emission production in commercial scales [1]. Microalgae convert CO_2_ into various bioresources for human domestic and industrial consumption [2]. Although microalgae have been used for thousands of years, the exploitation of the biological diversity of microalgae and their application in human nutrition is constrained by regulations. Up to now, only a few taxa are utilized for human consumption while *Spirulina* and *Chlorella* dominate the microalgal market [3].

The plant nature enables *Chlorella* as an excellent model organism to investigate the photosynthesis mechanisms and CO_2_ assimilation (Calvin–Benson cycle) from 1940s. Since 1950s, *Chlorella* spp. have been cultivated in huge quantities to meet the growing demand for alternative protein sources due to their short life span, robust environmental tolerance, and high content of proteins [4]. They are used as a food substitute for humans and are widely produced as health food in USA, Germany, China, Japan, and several other Asian countries [5]. Industrial *Chlorella* spp. include both freshwater and marine strains. The use of marine *Chlorella* strain in industrial systems is attractive due to the ever-expanding demand for fresh water, which is estimated to increase ~ 30% by 2050 [6]. Thus marine *Chlorella* strains have particular promise because they can grow on non-arable land and utilize saline water supplies.

We previously isolated a marine *Chlorella* strain MEM25 (hereafter MEM25) with a robust growth in a wide range of salinities, temperatures, and light intensities. Evaluation of the economic viability and performance of different scale cultivation system designs (photobioreactors and open race ponds; from 500 L to 60,000 L) using seawater showed the stable and robust characteristics of MEM25 across different production system designs and various spatial and temporal scales. It produces high amounts of proteins and polyunsaturated fatty acids in various conditions which underpins the nutritional merits of MEM25 as food additives [4]. In addition, a high-quality genome sequence of MEM25 reveals a compact genome structure (~ 50 M) and versatile metabolic pathways (*unpublished*). To improve the food feature of MEM25, chemical mutagenesis was employed to create a mutant library where a serial of mutant strains have been screened. In specific, a mutant shows a high CO_2_ capture capacity and increased levels of health beneficial metabolites and the antioxidant capacity (Gu et al., Biotechnology for Biofuels, Artificial switches induce the bespoke production of functional compounds in marine microalgae *Chlorella *by neutralizing CO_2_, in press). Moreover, MEM25 demonstrates a potential for a closed-loop circular bioeconomy by reclamating high-salinity seafood processing wastewater and the producing valuable bioproducts (Chen et al., Biomass & Bioenergy, Halophilic microalga-based circular economy producing functional food by reclaiming high-salinity seafood processing sewage, in press). Therefore, MEM25 is advantageous as a model organism for both basic research and commercial application.

To identify and improve species that naturally produce valuable compounds, transformation protocols have been created for common laboratory model algae, such as *Chlamydomonas reinhardtii* and *Phaeodactylum tricornutum*. However, relatively low biomass production rates in most of these strains have kept them from becoming industrially relevant. Genetic modifications have been utilized for *Chlorella* strains, but are largely via empirical approaches [7]. These earlier attempts entirely employed freshwater microalgae as models and the transformation methods reported have not been duplicated in the same strain or the same group. Possible reason is that these cases are largely relied on the use of foreign promoters [8–11] or a loss of transgenes in a majority of the transformants after a couple of months of cultivation. It indicates that the transgene had not truly integrated into the genome [12]. Or otherwise, current methods generally manipulate two genes (including a marker gene) at the maximal with relatively low transformation efficiency which limits its application [13].

To identify gene function and improve industrial properties, here we showed that the MEM25 transformants could be obtained in a relatively shorter period than that of the available model marine microalgae, such as *P. tricornutum* and *Nannochloropsis oceanica* [14]. Its mixotrophic capacity (that is MEM25 could use acetic acid as a carbon source) further reduces the screening time which is comparable to that of *Saccharomyces cerevisiae*. The transgene is integrated into the genome and can be successfully inherited for more than two years, the longest for *Chlorella* spp. as far as we know. The development of a MEM25 transformation method, in combination with the complete genome, will greatly facilitate more comprehensive mechanism studies and provides possibilities to use this species as chassis for synthetic biology to produce value-added compounds with mutual advantage in neutralizing CO_2_ in commercial scales.

## Materials and methods

### Strain and culture conditions

*Chlorella* sp. MEM25 was initially isolated from the inland saline water of Hainan Island and preserved in the State Key Laboratory of Marine Resource Utilization in South China Sea, Hainan University (Hainan, China). It is maintained in enriched F2 cultures with a salinity of 70‰, at an ambient temperature of 30 °C, with light:dark cycles (16:8) under light intensities approximately 200 μmol·photons·m^−2^·s^−1^ [4].

### Sensitivity test to antibiotics

Antibiotics sensitivity of MEM25 was conducted using both liquid and solid media. Algal cells in logarithmic phase in mid-logarithmic phase were inoculated into liquid medium in triplicates at a low initial density (OD_750_ = 0.1). The cultures were treated with different concentrations (from 0 to 1000 μg·mL^−1^) of a serial of antibiotics including kanamycin, ampicillin, hygromycin B, cephalosporin, neomycin sulfate, streptomycin, spectinomycin, gentamicin, zeocin, G418, chloramphenicol, and basta. Growth of MEM25 was monitored by detecting the turbidity at indicated intervals. For the one displaying inhibitory effects on cells, the minimum inhibitory concentration of individual antibiotics was determined with more concentration gradients. Meanwhile, mid-logarithmic phase algal cells were collected and washed with axenic water. The precipitation was resuspended to a concentration of approximately 1 × 10^8^ cells·mL^−1^. Aliquots of cells were transferred to agar plates in the presence or absence of varying concentrations of antibiotics. For chloramphenicol, ethanol was used as the control, and the experiment was performed in triplicate. After inoculation with MEM25, the plates were incubated at 30 °C and scored for growth visually.

### Promoter identification and isolation

We used RNA-sequencing (RNA-seq) data to find genes showing constantly high expression levels. Mid-logarithmic phase algal cells were transferred into fresh medium at 30 °C with a light intensity of 200 μmol·photons·m^−2^ s^−1^ for 3 h and 24 h. Aliquots of cells for transcript analysis were collected immediately and the total RNA of the algal cells was prepared using an RNA miniprep kit (Promega, China) and the concentrations were measured using the NanoDrop 2000 spectrophotometer (ThermoFisher, USA). Libraries were constructed and then sequenced using the HiSeq 2500 (Illumina), which generated more than 20 million read pairs per sample. Raw reads containing adapter, poly-N, and low-quality reads were filtered, and the effective data were mapped with the MEM25 reference genome using Hisat2 where more than 96% clean reads were mapped. The abundance of the transcripts was estimated using FeatureCounts where FPKM (Fragments per Kilo bases per Million reads) was shown. Three biologically independent sets of samples were prepared. As we cannot make an arbitrary assessment of all promoters, we applied two criterions to select viable promoters. First, promoters of the genes showing the constantly highest average expression level, that is the average FPKM values of all time points of the biological replicates were highest; Second, the gene expression variability across all three replicates and two time points were the least. The TOP 30 differentially expressed genes were based on their temporal expression patterns were chosen for further assessment. The featured modules in promoters were analyzed at PlantCARE (http://bioinformatics.psb.ugent.be/webtools/plantcare/html/). The genes retrieved are listed in Additional file 2.

### Construction of MEM25 expression vector pMEM-CP1

The MEM25 expression vector pMEM-CP1 was designed as described in the Results section. Putative promoter sequences were amplified from genomic DNA and validated by Sanger sequencing. Primers used are listed in Additional file 1: Table S1. Codon-optimized *hyg*, *mCherry* and *gfp* were used for the detection of transformants. The selection marker *ble*-*mCherry* fusion gene and *gfp* were driven by promoter regions of gene 3843 and 8657, respectively. Their terminators are 3’-untranslated regions of gene 8657 and 8655, respectively. For expression of antibacterial peptide MSI99, a vector pMEM-CP1-MSI99 was constructed where the foot-and-mouth-disease-virus 2A self-cleavage peptide (2A) was employed to transcriptionally fuse to antibiotic resistance gene *ble*, *MSI-99*, and *mCherry* genes (*Ble*-*2A*-*MSI99*-*2A*-*mCherry* fusion).

### Transformation of *Chlorella* sp. MEM25 by electroporation

*Chlorella* cells at logarithmic growth stage were harvested by centrifugation (4 °C, 5000 rpm, 5 min). The *Chlorella* pellet was washed twice with frozen sterile ddH_2_O. After removing the supernatant, the algae cells were suspended to a final concentration of 2 × 10^8^ cells·mL^−1^. For each electroporation, 200 μL *Chlorella* cells suspension and 3 μg linear DNA fragment (enzymatic products) were placed in a precooled 0.2 cm sterile electroporation cuvette. Meanwhile, the algae cells suspension without linear DNA fragment was involved as negative control. Electroporation was performed using a Gene Pulser Xcell (Bio-Rad) apparatus with 50 μF capacitance, 500 Ohm shunt resistance, and field strength at 2.0, 2.5, 3.0, 3.25, 3.5, 4.0, 4.5, 5.0, and 5.5 kV cm^−1^. After electroporation, the cells mixture was transferred to F2 medium and recovered overnight, and then spayed on agar plates containing indicated concentration of antibiotics.

### PCR detection of* Chlorella* transformants

Transformant colonies appeared after approximately 5 days and were typically transferred after 10 days to liquid medium containing 10 μg mL^−1^ zeocin. DNeasy Plant Mini Kit was utilized to extract genomic DNA of transforms after a week. PCR amplification of genomic DNA was performed using specific primers 3843up F1 and 8655down T6 (Additional file 1: Table S1). The PCR contained a 1 X PCR Ex-Taq buffer (Mg^2+^ plus), 0.2 mM of each dNTP, 0.25 μM of each primer, and 1 U of Ex-Taq (Takara, China) per 25 μl reaction system. Amplification conditions were as follows: an initial 94 °C, 5 min, followed by 30 cycles of 94 °C, 30 s, 60 °C, 30 s, 72 °C, 1 kb/min, and a final extension at 72 °C for 7 min [15]. The PCR fragments of the expected length were purified (Omega, China) and sequenced (Sangon, China).

### Fluorescence detection of *mGFP* and* mCherry* gene expression

Transformants grown in zeocin-resistant agar medium were used for fluorescence analysis, and wild-type MEM25 cells were used as negative control. An Olympus BX51 microscope (Olympus, Japan) with epifluorescence and differential interference contrast (DIC) optics was used (GFP: excitation 488 nm, emission 520 nm; mCherry: excitation 587 nm, emission 610 nm) [16, 17].

## Results

### Antibiotics sensitivity of *Chlorella* sp. MEM25

**Fig. 1:**
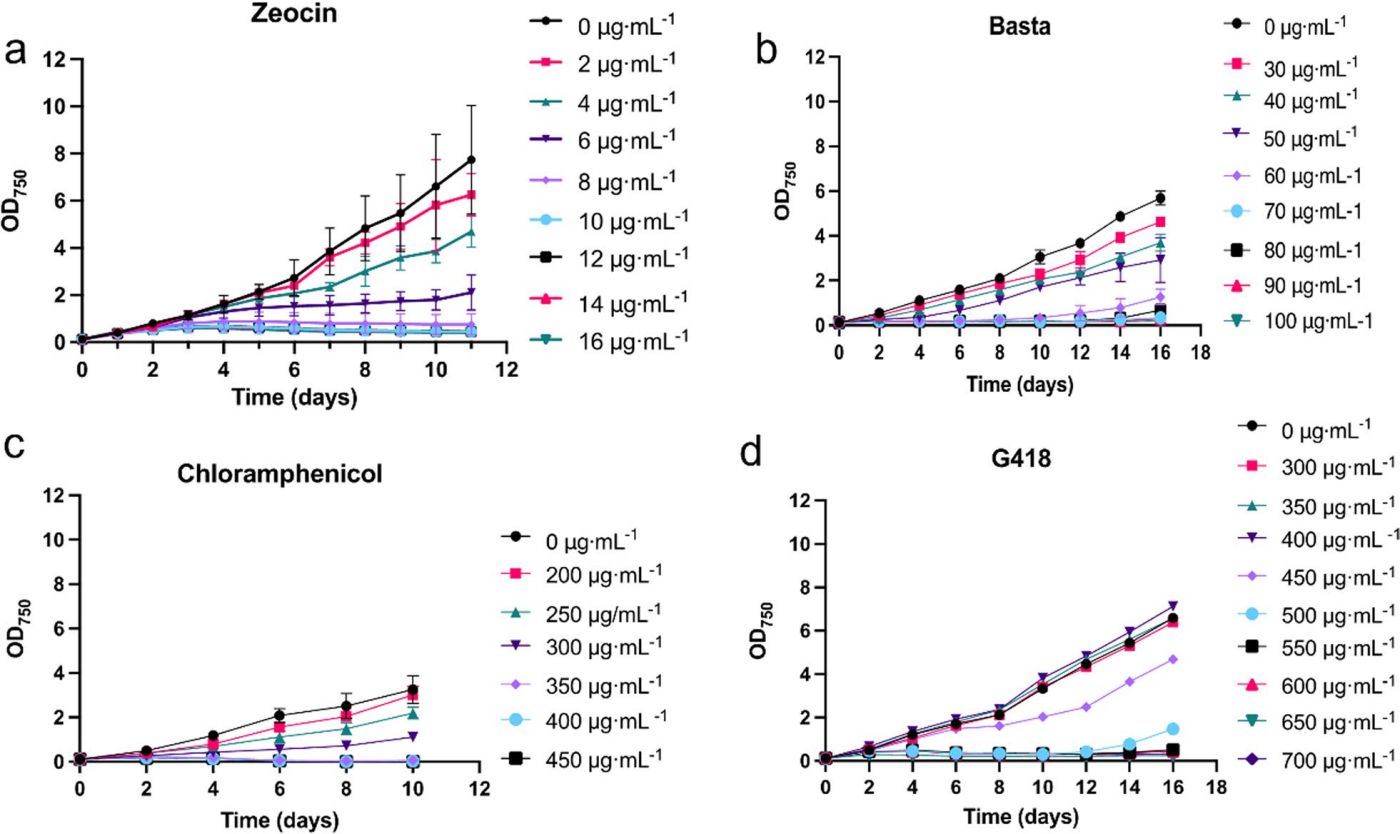
Antibiotics sensitivity of *Chlorella* sp. MEM25. The sensitivity of *Chlorella* sp. MEM25 to zeocin (**a)**, basta (**b)**, chloramphenicol (**c)** and G418 (**d)** were characterized in liquid F2 medium. Data are represented as mean value (*n* = 3 biologically independent samples)

To identify proper antibiotics with appropriate concentration with cytotoxic effect on MEM25, wild-type MEM25 cells were subjected to different concentrations of a serial of antibiotics including gentamicin, bleomycin, G418, chloramphenicol, basta, zeocin, kanamycin, ampicillin, cephalosporin, hygromycin B, neomycin sulfate, spectinomycin, and streptomycin. MEM25 was not susceptible to kanamycin, ampicillin, cephalosporin, and hygromycin B in the tested concentrations ranged from 0 to 1000 μg mL^−1^. Meanwhile, MEM25 is moderately sensitive to neomycin sulfate, spectinomycin, streptomycin, and gentamicin (Additional file 1: Figure S1). In contrast, zeocin, basta, chloramphenicol, and G418 displayed a dose-dependent inhibitory effect on MEM25 growth (Fig. 1). A week incubation with 8 μg mL^−1^ zeocin was sufficient to inhibit the growth of MEM25 (Fig. 1a) while basta (80 μg mL^−1^; Fig. 1b), chloramphenicol (350 μg mL^−1^; Fig. 1c), and G418 (550 μg mL^−1^; Fig. 1d) also significantly compromise the algal growth.

### Exogenous regulatory elements facilitate *ble*-expression in *Chlorella* sp. MEM25

**Fig. 2:**
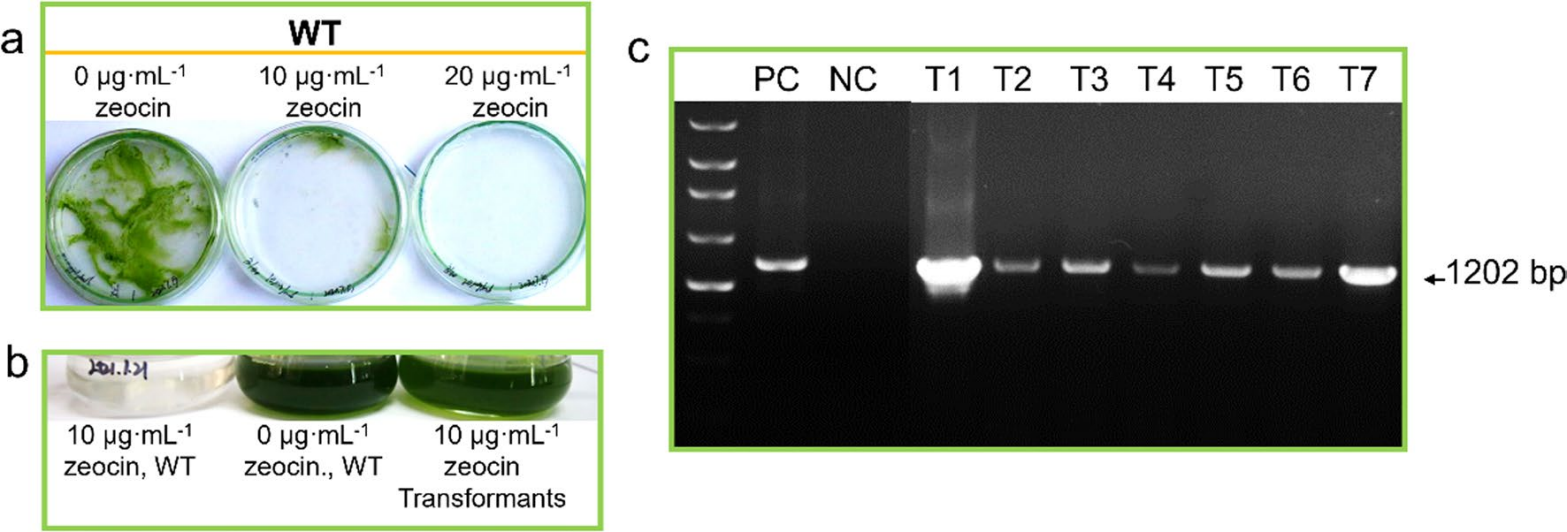
Screening and validation of *Chlorella* sp. MEM25 transformants. **a** Sensitivity of *Chlorella* sp. MEM25 to zeocin in solid F2. **b** Growth performance of pMS188 transformants after five generations of subculture. **c** PCR amplification of the genomic DNA of algal transformants. Arrow indicates the PCR product with expected size of 1202 bp. *PC* positive control, *NC* negative control, *T1-T7* transformants

To examine the validity the selective pressure, transformation experiments were performed using the pMS188 vector containing zeocin resistance gene *ble* driven by the *Chlamydomonas* HSP70A-RBCS2 promoter [18]. To further confirm the selection pressure, wild-type MEM25 was sprayed on solid F2 supplemented with zeocin, basta, chloramphenicol, and G418 with concentration gradients. Although the growth of MEM25 was compromised by these antibiotics, relatively higher concentrations were required for the selection on solid culture compared with that of liquid culture. For example, the algae could survive on F2 plates with as high as 10 μg mL^−1^ zeocin (Fig. 2a). A serial of voltage gradients were applied including 400 V, 500 V, 600 V, 650 V, 700 V, 800 V, 900 V, and 1000 V. Among all voltages, the range from 600 to 700 V achieved relatively stable delivery of vectors into the algal cells (i.e., peak transformation efficiency) (Additional file 1: Table S2). After the pulse, the algal cells were cultured on solid plate containing 20 μg mL^−1^ zeocin in continuous light at 25 ℃. Colonies appeared after approximately a week and were then transferred into liquid medium with 10 μg mL^−1^ zeocin (Fig. 2b). The colonies were typically confirmed after 10 days by PCR using specific primers of *ble* expression cassette (HSP70A-RBCS2-ble; Fig. 2c). The purified PCR products were validated as *ble* by sequencing. Therefore, the portfolio of *ble* gene and zeocin is competent in selection of MEM25 transformants.

### Design and expression foreign genes using native *cis* elements

To design expression vectors, *Chlorella* sp. MEM25 genome and mRNA-Seq-based transcriptome datasets were used to isolate *cis* elements. To maximize the coverage of transcripts sampled, the transcriptomic dynamics of MEM25 was monitored for 24 h (two life cycles). The experiment generated six high-quality transcriptome data sets with high reproducibility among the three biological replicates at each time point (Spearman correlation > 0.99). The transcript abundance (TA) of each gene at a particular time point was determined based on FPKM value (Fragments Per Kilobase of exon model per Million mapped fragments). An average FPKM value of 59.1 was obtained for all the genes at the two time points. TA of each gene was sorted according to FPKM values. Genes with an average FPKM value among the top 30 at the two time points were nominated as TOP 30 (Additional file 2). An average FPKM value of 4419 was obtained for the 30 genes.

Among TOP 30, the 5’-UTRs of genes P3843 and P8657 were selected as promoters and the 3’-UTRs of gene P8655 was selected as terminator while the transcriptional levels of the three genes were higher than the average value of all detected genes (Additional file 1: Table S3). For each of these genes, we analyzed the presence and location of the core elements known in eukaryotes [19] where the key regulatory elements were detected in all the three genes. However, the complexity of biology made it difficult to predict the extent to which such promoters will functionalize. Therefore, we further explored the in vivo activities of selected elements.

**Fig. 3:**
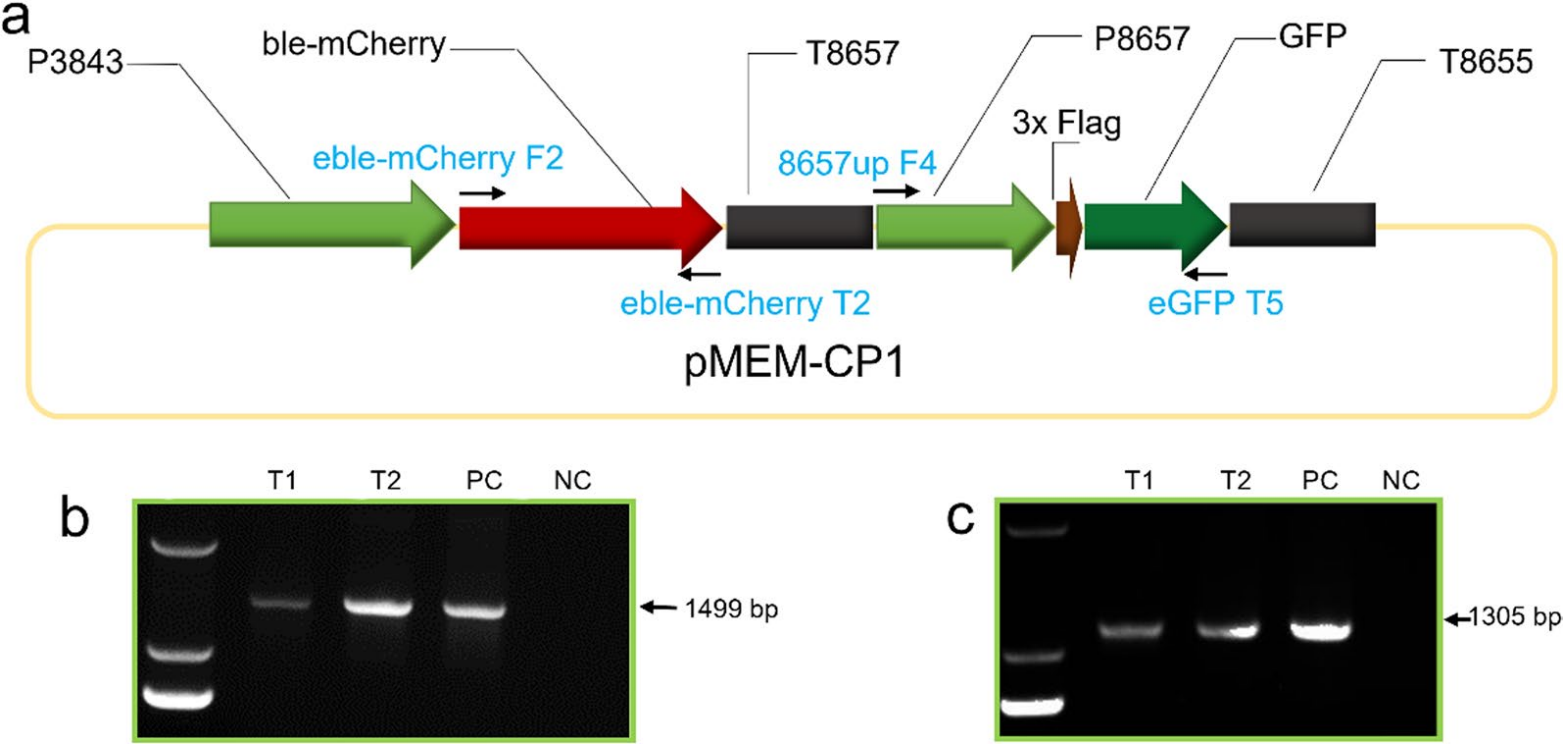
Design and expression foreign genes using native *cis* elements. **a** Map of the vector pMEM-CP1. Primers used for PCR amplification of the eGFP fragment and the eBle-mCherry fragment are shown. **b** PCR amplification of the fragment harboring eGFP in the genome of transformants. Arrow indicates the PCR product with expected size of 1499 bp. **c** PCR amplification of the *eBle*-*mCherry* fragment in the genome of transformants. Arrow indicates the PCR product with expected size of 1305 bp. *PC* positive control, *NC* negative control, *T1-T2* transformants

A fluorescent ble-mCherry fusion protein was designed to facilitate visualization of protein expression driven by P3843 (Fig. 3a). To realize multiple-gene expression, a separate green fluorescent protein (GFP) expressing cassette with P8657 was designed (Fig. 3a). Two pair of primers 8657up-F4/eGFP-T5 and eble-mCherry-F2/eble-mCherry-R2 were used to retrieve GFP and ble-mCherry cassettes, respectively (Fig. 3a and Additional file 1: Table S1). The integration of individual expression cassette was validated by amplification of genomic DNA and confirmed by sequencing (the eGFP fragment, Fig. 3b; the eBle-mCherry fragment, Fig. 3c). Voltage with a value of 700 V was used for electroporation. The efficiency of the transformations was strongly affected by the promoter, and was greatly improved by the use of endogenous promoters. The highest efficiency achieved (331 CFU/μg DNA; the highest among available approaches for *Chlorella* transformation [11, 20–23]), is considerably higher than that observed with the transformations of *Chlamydomonas* promoter (i.e., pMS188 vector; 232 CFU/μg DNA).

### Co-expression of multiple marker genes in engineered cells

**Fig. 4:**
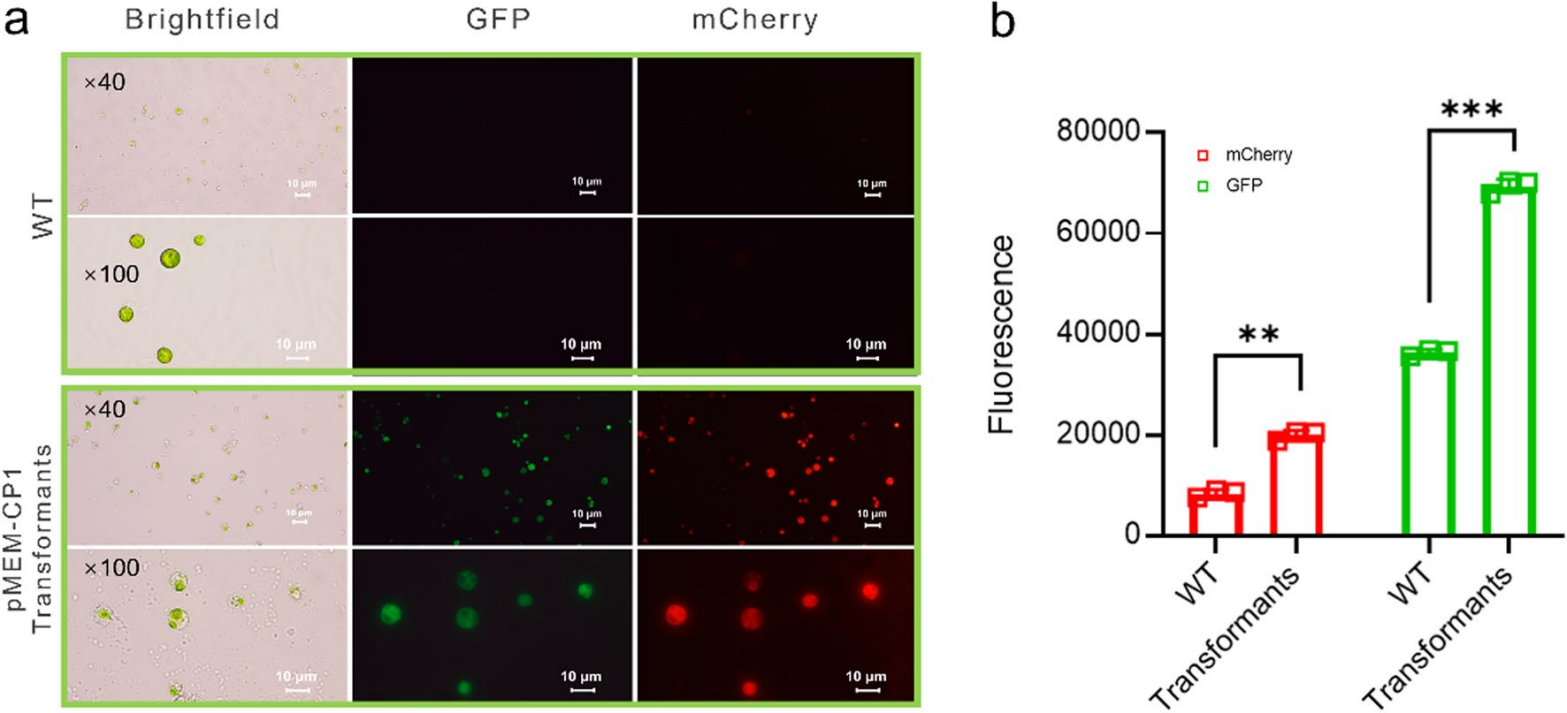
Co-expression of multiple genes in the *Chlorella* sp. MEM25. **a** Live-cell fluorescence microscopy of the GFP/BLE-expressing strains. Live cells were plated on agar pads and subjected to fluorescence microscopy. Images were collected using the optimal filters for each fluorescent protein (GFP: excitation 488 nm, emission 520 nm; mCherry: excitation 587 nm, emission 610 nm). For each fluorescent image shown, wild-type images were acquired using identical acquisition settings. Visible light microscope images are also shown. The fluorescent protein and respective WT images were processed identically. Scale bar = 10 µm. **b** Comparison of GFP or mCherry fluorescence between wild-type and transformant cells. Data are represented as mean ± SD (n = 3 biologically independent samples). *Significant change (**, *P* ≤ 0.05; ***, *P* ≤ 0.001. one-sided Student’s t test) versus the WT

The proper in vivo functioning of each of these expression cassettes in MEM25 was validated individually. Specifically, transcription and translation of the *ble* gene were verified by the screening pressure of 20 μg mL^−1^ zeocin. Moreover, live-cell fluorescence microscopy confirmed that the ble-mCherry protein was transcribed and efficiently processed to yield the mature protein (i.e., red fluorescence signals) in *Chlorella* sp. MEM25 (Fig. 4a). The in vivo expression of GFP driven by the P8657 promote was validated by detection the green fluorescence with an excitation at 488 nm (Fig. 4a). Despite a considerable background fluorescence for unknown reasons, fluorescent measurement using spectrophotometer further confirmed the expression of both mCherry and GFP. The fluorescence values of both in the transformants were twice as high as those of wild-type MEM25 (Fig. 4b). Therefore, all three genes were expressed properly in the transformants whereas WT can’t survive on zeocin plates and showed neither mCherry nor GFP fluorescence. Furthermore, for the engineered strains, we have preserved and inoculated twice a month from March 2020. The strains can survive on antibiotic plats and the fluorescence of both mCherry and GFP was observed at the end of 2022, suggesting that the transgene is integrated into the genome and can be successfully inherited for more than two years.

### Optimization of the transformant screening

To shorten the screening time of transformants, we developed a protocol to increase the growth rate and accelerate positive colony identification. After the electroporation, MEM25 was sprayed on F2 plates with various concentrations of acetic acid. Plates were observed regularly until the colonies were visible. The period from the cell spraying to the appearance of algal colonies was dramatically shortened by the addition of 1 mg mL^−1^ acetic acid (from two weeks to a week). It is time-saving compared with that of *P. tricornutum* (approximately 20 days) [24] and *N. oceanica* (approximately 15 days) [14] and comparable to that of *C. reinhardtii* (is approximately 7 days) [25].

To identify positive strains quickly and easily, a direct colony PCR protocol was developed. Colonies were typically transferred and suspended in 50 μL Na-EDTA (20 mM) after 4 days. The mixture was incubated at 98 °C for 5 min for cell disruption after a gentle vortex. The cell debris was spun down at 12,000 g for 1 min and 1 μL supernatant was used as template for a 25 μL PCR reaction. The direct colony PCR protocol for MEM25 was highly effective and time saving and could generally obtain 76% successes for amplification of internal genes within approximately 1 h. Therefore, a combination of the addition with acetic acid and the direct colony PCR, a normal round of positive strain screening after electrophoresis could be reduced to within a week.

### Expression of an antibacterial peptide and genetic stability of transgenic hallmarks

**Fig. 5:**
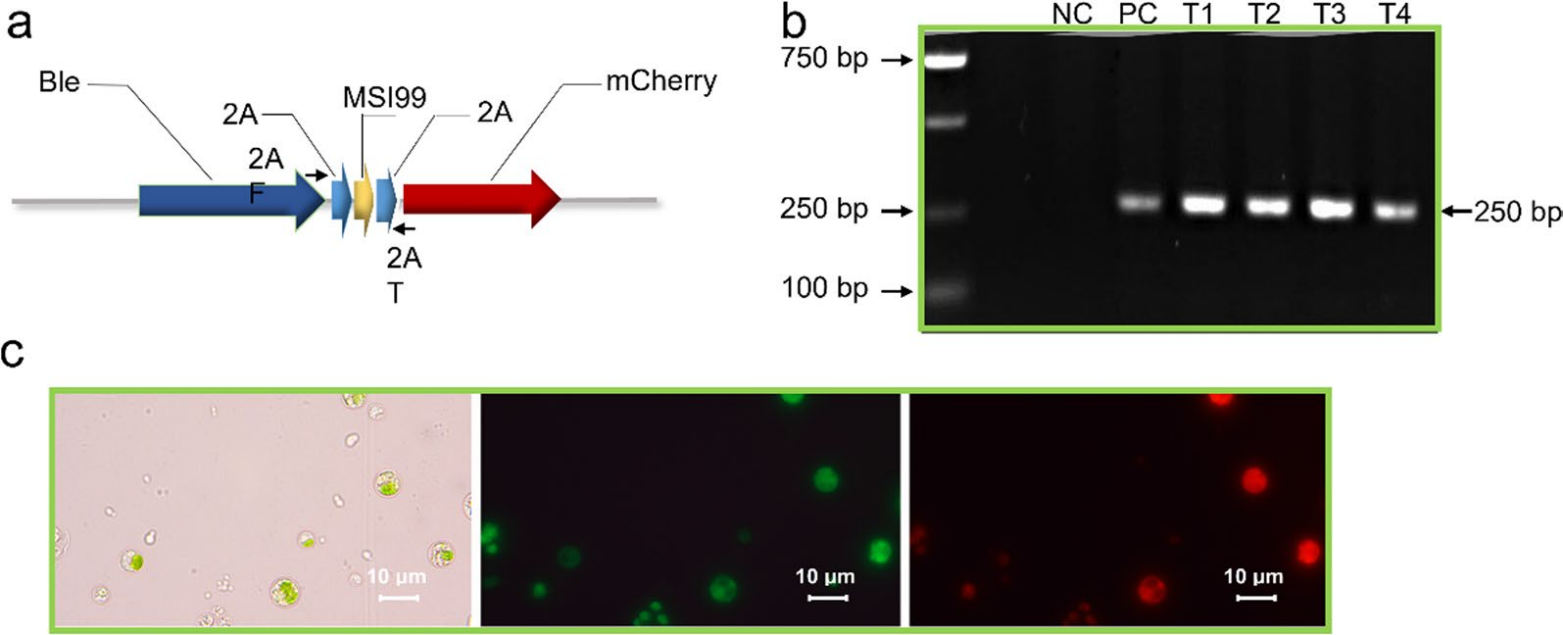
Genetic stability of transgenic hallmarks. **a** The core region of the overexpression vector pMEM01-Ble-2A-MSI99-2A-mCherry. **b** PCR validation of the integration of MSI-99 in transformants. **c** Live-cell fluorescence microscopy of the transformants expressing both GFP and mCherry

The antimicrobial peptide MSI-99, an analog of magainin 2, is a defense peptide secreted from the skin of the African clawed frog (*Xenopus laevis*). A synthetic substitution analogue of MSI-99 has been employed to improve the disease resistance of crops [26]. An expression cassette harboring *ble*, *MSI-99*, and *mCherry* genes was designed where 2A was employed to transcriptionally fuse the three genes (*Ble*-*2A*-*MSI-99*-*2A*-*mCherry* fusion) (Fig. 5a). The transcription and translation of the *ble* gene were verified by selecting transformants on F2 plates supplemented with zeocin. Live-cell fluorescence microscopy of the *Chlorella* transformants confirmed that the mCherry protein was transcribed and efficiently processed to yield the mature protein, as revealed via the red fluorescence signals. The antibacterial activity against *Escherichia coli* and fish-related pathogens (e.g., *Vibrio* spp. and *Staphylococcus aureus*) cannot be validated from the transformant extracts, potentially due to the low protein level resulted from the cellular toxicity of MSI-99. Nevertheless, as *MSI-99* locates between *Ble* and *mCherry* genes in the expression cassette, it suggests a proper in vivo expression of the MSI-99 peptide. PCR and sequencing confirmed the MSI-99 integration into the genome of *Chlorella* sp. MEM25 (Fig. 5b).

We then tested whether the transgene can be stably inherited through generations. The transgenic lines were inoculated into liquid culture without selection pressure where they grew robustly after five generations of subculture. The growth of wild-type MEM25 was set as control. Afterwards, the transformants were sprayed on F2 plates containing zeocin where colonies could be observed after four days. Moreover, both mCherry and GFP fluorescence can be detected (Fig. 5c), indicating that the transgene and its expression was successfully endowed into the next generation. Therefore, four genes could be expressed simultaneously in the marine *Chlorella* sp. using this engineering system.

## Conclusion and discussion

The ability to site microalgal systems on non-arable land and to use saline water is an increasingly important consideration for policymakers as the global population, with its need for food, fuel, chemical feedstocks, and fresh water, is forecast to increase from 7.2 billion to 9.6 billion by 2050 [27–29]. Although engineering systems of marine species *P. tricornutum* and *N. oceanica* are available, the biomass production rates of both species are incomparable to most *Chlorella* strains, particularly if the cultivation locates in (sub)tropical areas. On the other hand, although locations around the equator do not necessarily produce the largest yields of microalgal biomass, they are typically considered to be optimum cultivation locations because of their annual temperature stability [30]. Therefore, the development of an engineering system for tropical marine *Chlorella* strains is of particular interest for research on microalga-based carbon neutralization and negative emission production in commercial scales.

Due to the demonstrated industrial potential, *Chlorella* spp. have been extensively explored as human nutritional food and cell factories to produce therapeutic proteins. However, all *Chlorella* strains that have been transformed are freshwater species, but the potential of using marine strains has not been probed. Moreover, the hallmarks of transgenes have only been inherited for several generations, potentially due to the events that the transgene had not truly integrated into the algal genome [12]. Here, the protocol described in this study successfully generated stable transformants in marine *Chlorella* strain MEM25 of which the production capacity has been demonstrated in industrial scale. It is the first example for the transformation of the marine *Chlorella* and the efficiency is the highest among available transformation approaches for either freshwater or marine *Chlorella* strains. Moreover, transformed colonies could be obtained within a week which is dramatically  time saving compared with the documented model species, such as *P. tricornutum* and *N. oceanica* [31]. Its mixotrophic capacity (that is MEM25 could use acetic acid as a carbon source) further reduces the screening time which is comparable to that of *Saccharomyces cerevisiae*.

Furthermore, we confirmed that the transgene can be successfully inherited for more than two years, the longest for engineered *Chlorella* spp. as far as we know. An additional merit of this method is that multiple genes have been expressed simultaneously in MEM25. In contrast, for available methods for *Chlorella* spp., they generally manipulate two genes (including a marker gene) at the maximal with relatively low transformation efficiency which limits its application [13]. The development of a MEM25 transformation method, in combination with the complete genome, will greatly facilitate more comprehensive studies of the mechanisms underpinning its high CO_2_ absorption. Although the protein accumulation is still not high, with further optimization, it opens a door to use this species as chassis for synthetic biology to produce therapeutic proteins with mutual advantage in neutralization of CO_2_ in commercial scales.

### Supplementary Information

**Additional file 1: Figure S1.** Antibiotics sensitivity of *Chlorella *sp. MEM25. **(a)** Sensitivity of *Chlorella* sp. MEM25 to kanamycin. **(b)** Sensitivity of *Chlorella* sp. MEM25 to ampicillin. **(c)** Sensitivity of *Chlorella* sp. MEM25 to cephalosporin. **(d)** Sensitivity of *Chlorella* sp. MEM25 to hygromycin B. **(e)** Sensitivity of *Chlorella* sp. MEM25 to neomycin sulfate. **(f)** Sensitivity of *Chlorella* sp. MEM25 to spectinomycin. **(g)** Sensitivity of *Chlorella* sp. MEM25 to streptomycin. **(h)** Sensitivity of *Chlorella* sp. MEM25 to gentamicin. **Table S1**. Primers used in this study. **Table S2**. Transformation efficiency under different voltage during electroporation *Chlorella* sp. MEM25. **Table S3**. Transcriptional levels of genes of which the promoters are used for vector construction in this study.

**Additional file 2: Dataset 1**. The mRNA-Seq-based transcriptome datasets of *Chlorella* sp. MEM25.

## Data Availability

All data generated or analyzed during this study are included in this published article and its supplementary information files.

## References

[1] Ashraf N, Ahmad F, Lu Y (2022). Synergy between microalgae and microbiome in polluted waters. Trends Microbiol.

[2] Zhou W (2022). Widespread sterol methyltransferase participates in the biosynthesis of both C4α- and C4β-methyl sterols. J Am Chem Soc.

[3] Pulz O (2001). Photobioreactors: production systems for phototrophic microorganisms. Appl Microbiol Biotechnol.

[4] Lu X (2021). Sustainable development of microalgal biotechnology in coastal zone for aquaculture and food. Sci Total Environ.

[5] Liu J, Chen F (2016). Biology and industrial applications of *Chlorella*: advances and prospects. Adv Biochem Eng/Biotechnol.

[6] Beddington J (2010). Food, energy, water and the climate: a perfect storm of global events. UK Govern Office Sci.

[7] Lu Y, Zhang X, Gu X, Lin H, Melis A (2021). Engineering microalgae: transition from empirical design to programmable cells. Critical Rev Biotechnol.

[8] Sharma PK, Goud VV, Yamamoto Y, Sahoo L (2021). Efficient *Agrobacterium tumefaciens*-mediated stable genetic transformation of green microalgae *Chlorella sorokiniana*. 3 Biotech.

[9] Kim J, Chang KS, Lee S, Jin E (2021). Establishment of a genome editing tool using CRISPR-Cas9 in *Chlorella vulgaris* UTEX395. Int J Mol Sci.

[10] Lin WR, Ng IS (2020). Development of CRISPR/Cas9 system in *Chlorella vulgaris* FSP-E to enhance lipid accumulation. Enzyme Microb Technol.

[11] Liu L (2013). Development of a new method for genetic transformation of the green alga Chlorella ellipsoidea. Mol Biotechnol.

[12] Vieler A (2012). Genome, functional gene annotation, and nuclear transformation of the heterokont oleaginous alga *Nannochloropsis oceanica* CCMP1779. PLoS Genet.

[13] Tokunaga S, Sanda S, Uraguchi Y, Nakagawa S, Sawayama S (2019). Overexpression of the DOF-type transcription factor enhances lipid synthesis in *Chlorella vulgaris*. Appl Biochem Biotechnol.

[14] Wang Q (2016). Genome editing of model oleaginous microalgae *Nannochloropsis* spp. by CRISPR/Cas9. Plant J.

[15] Gan Q (2017). A customized contamination controlling approach for culturing oleaginous *Nannochloropsis oceanica*. Algal Res.

[16] Gan Q, Jiang J, Han X, Wang S, Lu Y (2018). Engineering the chloroplast genome of oleaginous marine microalga *Nannochloropsis oceanica*. Front Plant Sci.

[17] Rasala BA (2013). Expanding the spectral palette of fluorescent proteins for the green microalga Chlamydomonas reinhardtii. Plant J.

[18] Strenkert D, Schmollinger S, Schroda M (2013). Heat shock factor 1 counteracts epigenetic silencing of nuclear transgenes in *Chlamydomonas reinhardtii*. Nucleic Acids Res.

[19] Juvengershon T, Hsu J, Theisen JWM, Kadonaga JT (2008). The RNA polymerase II core promoter—the gateway to transcription. Curr Opin Cell Biol.

[20] Gadamchetty P, Mullapudi PLV, Sanagala R, Markandan M, Polumetla AK (2019). Genetic transformation of *Chlorella vulgaris* mediated by HIV-TAT peptide. 3 Biotech.

[21] Ji L, Fan J (2020). Electroporation procedures for genetic modification of green algae (Chlorella spp.). Methods Mole Biol.

[22] Zhang J (2014). Overexpression of the soybean transcription factor GmDof4 significantly enhances the lipid content of *Chlorella ellipsoidea*. Biotechnol Biofuels.

[23] Run C (2016). Stable nuclear transformation of the industrial alga *Chlorella pyrenoidosa*. Algal Res.

[24] Cui Y, Zhao J, Wang Y, Qin S, Lu Y (2018). Characterization and engineering of a dual-function diacylglycerol acyltransferase in the oleaginous marine diatom *Phaeodactylum tricornutum*. Biotechnol Biofuels.

[25] Kim J, Lee S, Baek K, Jin E (2020). Site-specific gene knock-out and on-site heterologous gene overexpression in *Chlamydomonas reinhardtii* via a CRISPR-Cas9-mediated knock-in method. Front Plant Sci.

[26] Chakrabarti A, Ganapathi TR, Mukherjee PK, Bapat VA (2003). MSI-99, a magainin analogue, imparts enhanced disease resistance in transgenic tobacco and banana. Planta.

[27] Gan Q (2017). Culture-free detection of crop pathogens at the single-cell level by micro-Raman spectroscopy. Adv Sci.

[28] Chen Z (2021). Isolation of a novel strain of Cyanobacterium sp. with good adaptation to extreme alkalinity and high polysaccharide yield. J Oceanol Limnol.

[29] Han X, Song X, Li F, Lu Y (2020). Improving lipid productivity by engineering a control-knob gene in the oleaginous microalga *Nannochloropsis oceanica*. Metabolic Eng Commun.

[30] Moody JW, McGinty CM, Quinn JC (2014). Global evaluation of biofuel potential from microalgae. Proc Natl Acad Sci.

[31] Lu Y (2021). Role of an ancient light-harvesting protein of PSI in light absorption and photoprotection. Nat Commun.

