# Supplementary material for: Engineering a marine microalga Chlorella sp. as the cell factory
Source: Biotechnol Biofuels Bioprod. 2023 Sep 7;16:133. doi: 10.1186/s13068-023-02384-2 (PMC10485975; doi:10.1186/s13068-023-02384-2)
Supplement: Supplementary file 1 — Additional file 1: Figure S1. Antibiotics sensitivity of Chlorella sp. MEM25. (a) Sensitivity of Chlorella sp. MEM25 to kanamycin. (b) Sensitivity of Chlorella sp. MEM25 to ampicillin. (c) Sensitivity of Chlorella sp. MEM25 to cephalosporin. (d) Sensitivity of Chlorella sp. MEM25 to hygromycin B. (e) Sensitivity of Chlorella sp. MEM25 to neomycin sulfate. (f) Sensitivity of Chlorella sp. MEM25 to spectinomycin. (g) Sensitivity of Chlorella sp. MEM25 to streptomycin. (h) Sensitivity of Chlorella sp. MEM25 to gentamicin. Table S1. Primers used in this study. Table S2. Transformation efficiency under different voltage during electroporation Chlorella sp. MEM25. Table S3. Transcriptional levels of genes of which the promoters are used for vector construction in this study. [file 13068_2023_2384_MOESM1_ESM.docx]

**
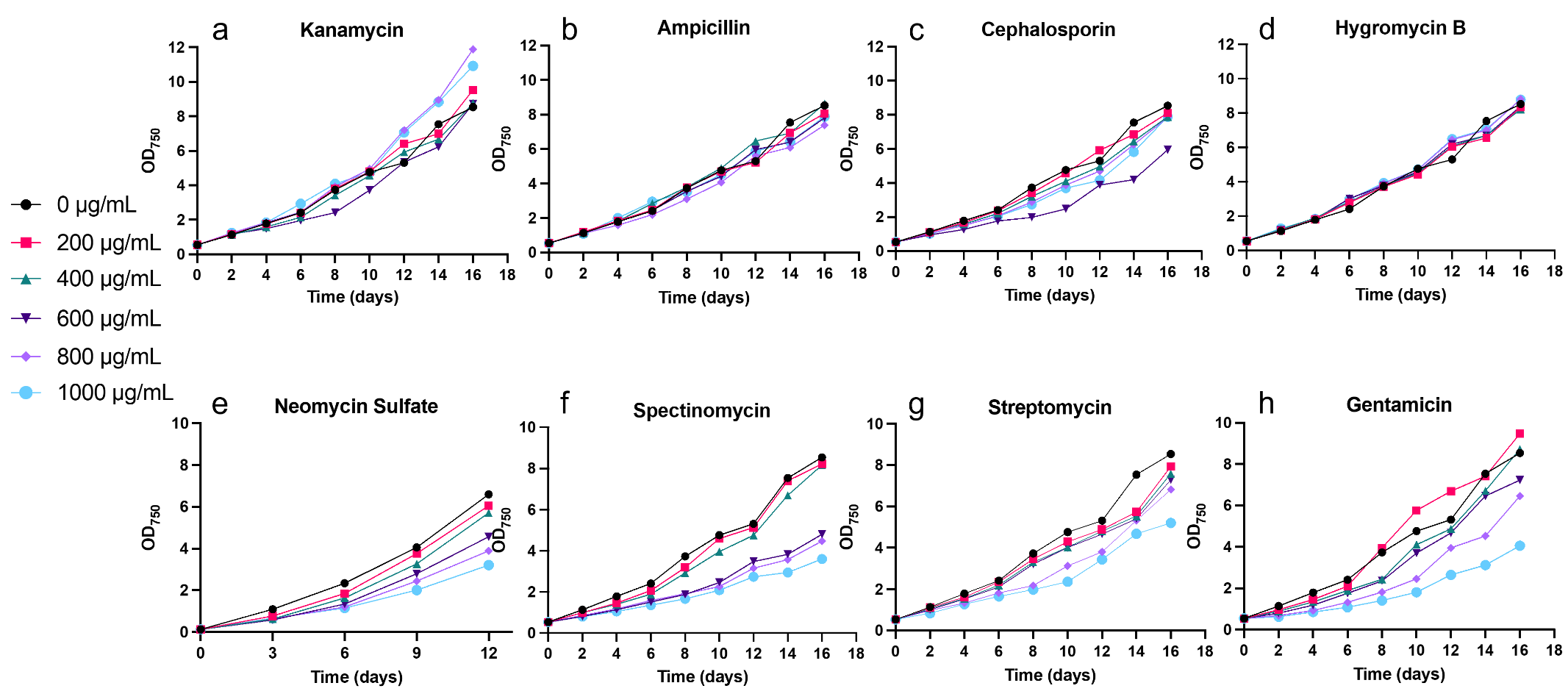
**

**Figure S1. Antibiotics sensitivity of *Chlorella* sp. MEM25. (a)** Sensitivity of *Chlorella* sp. MEM25 to kanamycin. **(b)** Sensitivity of *Chlorella* sp. MEM25 to ampicillin. **(c)** Sensitivity of *Chlorella* sp. MEM25 to cephalosporin. **(d)** Sensitivity of *Chlorella* sp. MEM25 to hygromycin B. **(e)** Sensitivity of *Chlorella* sp. MEM25 to neomycin sulfate. **(f)** Sensitivity of *Chlorella* sp. MEM25 to spectinomycin. **(g)** Sensitivity of *Chlorella* sp. MEM25 to streptomycin. **(h)** Sensitivity of *Chlorella* sp. MEM25 to gentamicin.

**Table S1.** Primers used in this study.

| Primers | Sequence（5′-3′） |
| --- | --- |
| 3843up F1 | GTACCGGGCCCCCCCTCGAGCACTCAGCAAGGAGTGGTCACAA |
| 3843up T1 | ACGGCGCTGGTCAACTTGGCCATCTCGAGAGCGAGTGCAACCATCTTGAC |
| eble-mCherry F2 | GTCAAGATGGTTGCACTCGCTCTCGAGATGGCCAAGTTGACCAGCGCCGT |
| eble-mCherry T2 | GCACAAGCTGCAAGCTGCTTACTGCAGTCACTTATAGAGTTCG |
| 8657down F3 | CGAACTCTATAAGTGACTGCAGTAAGCAGCTTGCAGCTTGTGC |
| 8657down T3 | GGCGGCCGCTCTAGAAAACGGATGCAGTGTGCCC |
| 8657up F4 | CTAGAGCGGCCGCCACCGCGGAGGAAAATTTGGACTCGGTGACG |
| 8657up T4 | TGTCGTGATCCTTGTAGTCCCCGTCGTGATCCTTGTAGTCCATCCGCGGGGCTATGTTTGTGTGTAAGTTGGGC |
| eGFP F5 | GACGGGGACTACAAGGATCACGACATCGACTACAAGGATGACGATGACAAGGAATTCGTGAGCAAGGGCGAGGA |
| eGFP T5 | CGCAAGTGCTCCCTTTCAGAATTCCTACTTGTACAGCTCGTCCATGCC |
| 8655down F6 | GGCATGGACGAGCTGTACAAGTAGGAATTCTGAAAGGGAGCACTTGCG |
| 8655down T6 | CGAATTGGAGCTCCATCAGACGTGGCAGGTCTGATC |
| 2A F | TCGTGGCCGAGGAGCAGGAC |
| 2A T | CTCTTCCCCTTTCGAAACCTGCAGA |

**Table S2.** Transformation efficiency under different voltage during electroporation of *Chlorella* sp. MEM25.

| **Voltages**  **(kV·cm^-1^)** | **Number of clones**  **（3 μg of DNA was transformed）** | **transformation efficiency**  **（CFU/μg DNA）** |
| --- | --- | --- |
| 2.0 | 0 | / |
| 2.5 | 0 | / |
| 3.0 | 286 | 0.95x10^2^ |
| 3.25 | 153 | 0.51x10^2^ |
| 3.5 | 552 | 1.84x10^2^ |
| 4.0 | 0 | / |
| 4.5 | 0 | / |
| 5.0 | 0 | / |
| 5.5 | 0 | / |

**Table S3.** Transcriptional levels of genes of which the promoters are used for vector construction in this study.

| **Gene** | **FPKM values** | |
| --- | --- | --- |
|  | **3h** | **24h** |
| P3843 | 9696.16±917.41 | 4526.91±303.15 |
| P8657 | 16449.80±1117.95 | 6694.22±744.78 |
| Average values  of all transcripts | 59.18±1.42 | 59.10±1.40 |
